# Changes in Social Network Size Are Associated With Cognitive Changes in the Oldest-Old

**DOI:** 10.3389/fpsyt.2020.00330

**Published:** 2020-05-04

**Authors:** Susanne Röhr, Margrit Löbner, Uta Gühne, Kathrin Heser, Luca Kleineidam, Michael Pentzek, Angela Fuchs, Marion Eisele, Hanna Kaduszkiewicz, Hans-Helmut König, Christian Brettschneider, Birgitt Wiese, Silke Mamone, Siegfried Weyerer, Jochen Werle, Horst Bickel, Dagmar Weeg, Wolfgang Maier, Martin Scherer, Michael Wagner, Steffi G. Riedel-Heller

**Affiliations:** ^1^Institute of Social Medicine, Occupational Health and Public Health (ISAP), Medical Faculty, University of Leipzig, Leipzig, Germany; ^2^Department for Neurodegenerative Diseases and Geriatric Psychiatry, University Hospital Bonn, Bonn, Germany; ^3^DZNE, German Center for Neurodegenerative Diseases, Bonn, Germany; ^4^Institute of General Practice, Medical Faculty, Heinrich-Heine-University Düsseldorf, Düsseldorf, Germany; ^5^Department of Primary Medical Care, Center for Psychosocial Medicine, University Medical Center Hamburg-Eppendorf, Hamburg, Germany; ^6^Institute of General Practice, University of Kiel, Kiel, Germany; ^7^Department of Health Economics and Health Services Research, Hamburg Center for Health Economics, University Medical Center Hamburg-Eppendorf, Hamburg, Germany; ^8^Work Group Medical Statistics and IT-Infrastructure, Institute for General Practice, Hannover Medical School, Hannover, Germany; ^9^Central Institute of Mental Health, Medical Faculty, Mannheim/Heidelberg University, Mannheim, Germany; ^10^Department of Psychiatry, Klinikum rechts der Isar, Technical University of Munich, Munich, Germany; ^11^Department of Psychiatry, University of Bonn, Bonn, Germany

**Keywords:** social network, social isolation, cognitive function, oldest-old, lifestyle, risk factor, cohort study, prevention

## Abstract

**Objectives:**

Social isolation is increasing in aging societies and several studies have shown a relation with worse cognition in old age. However, less is known about the association in the oldest-old (85+); the group that is at highest risk for both social isolation *and* dementia.

**Methods:**

Analyses were based on follow-up 5 to 9 of the longitudinal German study on aging, cognition, and dementia in primary care patients (AgeCoDe) and the study on needs, health service use, costs, and health-related quality of life in a large sample of oldest-old primary care patients (AgeQualiDe), a multi-center population-based prospective cohort study. Measurements included the Lubben Social Network Scale (LSNS-6), with a score below 12 indicating social isolation, as well as the Mini-Mental Status Examination (MMSE) as an indicator of cognitive function.

**Results:**

Dementia-free study participants (n = 942) were *M* = 86.4 (*SD* = 3.0) years old at observation onset, 68.2% were women. One third (32.3%) of them were socially isolated. Adjusted linear hybrid mixed effects models revealed significantly lower cognitive function in individuals with smaller social networks (*β* = 0.5, 95% CI = 0.3–0.7, *p* < .001). Moreover, changes in an individual’s social network size were significantly associated with cognitive changes over time (*β* = 0.2, 95% CI = 0.1–0.4, *p* = .003), indicating worse cognitive function with shrinking social networks.

**Conclusion:**

Social isolation is highly prevalent among oldest-old individuals, being a risk factor for decreases in cognitive function. Consequently, it is important to maintain a socially active lifestyle into very old age. Likewise, this calls for effective ways to prevent social isolation.

## Introduction

Social isolation is highly prevalent among older individuals (10–43%) (1). Social network size tends to decrease with age (2). Moreover, social isolation has become more prevalent over the past decades, suggesting a secular trend toward shrinking and more fragile social networks (3). Considering that population aging is rapidly increasing the proportion of older individuals worldwide, social isolation may constitute an even greater burden to societies in the near future.

The epidemiological development of social isolation is worrying because of its negative effects on physical health and mental health (4, 5). It is also associated with increased all-cause-mortality (6). Social isolation is an objective state where an individual has minimal contact with others and low levels of engagement in the community (7, 8). It constitutes a chronically stressful condition that accelerates aging (9). Conversely, this means that social integration, hence being part of a meaningful social network, is a fundamental need, crucial for well-being and survival. Theoretically, social integration is understood to have (1) a buffering effect through being a resource in times of stress, e.g., during illness or loss, and (2) to have a main effect on well-being as it provides regular positive experiences and a stable role in the community (10). Thus, social isolation is the opposite of social integration. Assessing social network size is a typical indicator for both, in which—depending on the instrument—established cutoffs differentiate between social isolation and social integration (11). As such, it is a quantitative measure that needs to be differentiated from qualitative aspects of evaluating one’s social network, most importantly loneliness. Social isolation describes a state of *being* lonely and loneliness refers to a poor subjective evaluation one own’s relationships, hence *feeling* lonely (12). Individuals can indeed feel lonely despite being integrated in a large social network and socially isolated individuals may not feel lonely at all (13).

As social isolation may unfold in higher morbidity and faster decline with aging, one area of interest in this regard is cognitive function and associated disorders such as cognitive impairment and dementia, which are among the leading causes of disability and dependency in old age (14). Studies predominantly show that social isolation is associated with cognitive decline (15, 16) and a higher likelihood to develop dementia (17, 18). In fact, social isolation is recognized among a set of specific modifiable risk factors for dementia (19). Three potential mechanisms may explain the adverse effect of social isolation on cognition: a) less physical activation through less social activity, b) less cognitive stimulation through limited social interaction, and c) less resources for positive emotions that may buffer stress (20).

While the relationship of social isolation and cognitive function in late life has been studied comprehensively (for reviews see: 21, 22), this is less the case for the segment of the oldest-old (85+). In their meta-analysis, Evans et al. (22) identified three longitudinal studies that addressed populations with a mean age well over 80 years: Bennett et al. (23) investigated social network in relation to Alzheimer’s disease pathology and cognitive function postmortem in 89 US individuals aged 84.3 years on average at baseline, suggesting that social network modified this relation in regard to some measures (tangles; semantic memory, working memory). In a sample of Chinese oldest-old (*M* = 83.6 years), being single and having less frequent contact with family members were associated with cognitive decline over 2 years (24). Brown et al. (25) reported that changes in social activity were related to changes in cognitive performance (i.e., reasoning, semantic knowledge, memory) over 8 years in the Swedish origins of variance in the old-old (OCTO)-Twin study, a sample of dementia-free individuals aged 80–85 years at baseline. As lifestyle associations with cognitive function and dementia may present differently in midlife *vs.* younger-old age *vs.* older-old age (19, 26), we aimed to add to the growing body of literature by investigating longitudinal effects of social network size, as an indicator of an individual’s degree of social isolation or social integration, on cognitive function in dementia-free oldest-old individuals.

Specifically, we aimed to disentangle effects of social network size *between* subjects and effects of changes in social network size *within* subjects on cognitive function, while accounting for confounders. Thus, we hypothesize that social network size is longitudinally associated with cognitive function, i.e., i) individuals with smaller social networks should have lower cognitive function than individuals with larger social networks, and ii) changes in an individual’s social network size should be longitudinally associated with changes in cognitive function, whereas decreases in social network size should be associated with decreases in cognitive function. Considering that social isolation and dementia are both highly prevalent among the oldest-old, our results should have important implications for public health.

## Materials and Methods

### Study Design

This study draws on the German study on aging, cognition, and dementia in primary care patients (AgeCoDe), a prospective longitudinal cohort study on mild cognitive impairment (MCI) and dementia, and its extension/continuation the study on needs, health service use, costs, and health-related quality of life in a large sample of oldest-old primary care patients (AgeQualiDe). The AgeCoDe/AgeQualiDe-study was conducted in six German cities (Bonn, Duesseldorf, Hamburg, Leipzig, Mannheim, Munich). Participants were recruited by 138 general practitioners (GP) between January 2003 and November 2004. Inclusion criteria were ≥75 years of age, no dementia, and at least one GP contact within the last year. Exclusion criteria comprised GP consultation at home only, nursing home residence, severe illness with an expected fatal outcome within 3 months, German language insufficiency, deaf- or blindness, and inability to provide informed consent.

Initially, 3,327 GP patients were investigated, of which 2,326 (69.9%) were women. In total, 113 (3.4%) individuals were excluded because of prevalent dementia (*n* = 70, 2.1%), age <75 years (*n* = 39, 1.2%), and incomplete assessments (*n* = 4, 0.1%). Finally, 3,214 individuals constituted the AgeCoDe/AgeQualiDe cohort. Nine follow-up assessments were scheduled every 1.5 years up to follow-up 7 and then every 10 months up to follow-up 9. Study details have been described elsewhere (27).

### Ethics

All participants provided written informed consent. The study protocol has been approved by the local ethic committees of all participating centers and complies with the guidelines of the Helsinki Declaration.

### Dataset and Analytical Sample

**Figure 1:**
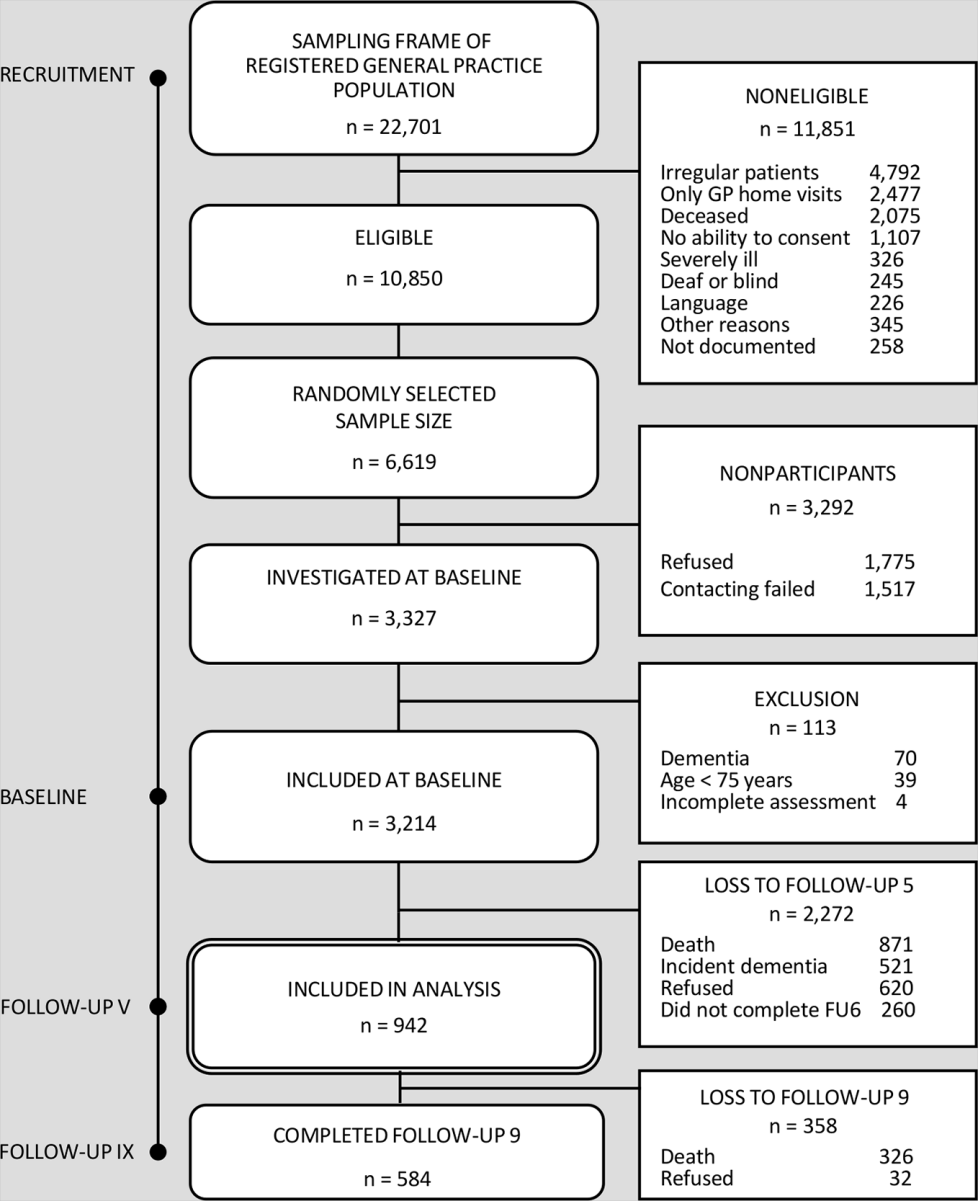
Flowchart - recruitment and analytical sample selection of the German study on aging, cognition, and dementia in primary care patients (AgeCoDe)/the study on needs, health service use, costs, and health-related quality of life in a large sample of oldest-old primary care patients (AgeQualiDe).

For the purpose of this study, we draw on data of all dementia-free study participants at follow-up 5 (first assessment wave, in which social network was assessed) of the AgeCoDe/AgeQualiDe study, who completed at least one further wave. These data were collected between September 2009 (beginning of follow-up 5) and November 2016 (end of follow-up 9), comprising a total of five assessment waves over 4.7 years. A total of *n* = 1,314 individuals participated in follow-up 5. We excluded *n* = 159 (12.1%) due to incident dementia, *n* = 171 (13.0%) due to missing follow-up data, *n* = 42 (3.2%) due to incomplete assessments in cognitive function, social network and sociodemographic variables, leading to an analytical sample of *n* = 942 (**Figure 1**).

### Assessments

Trained psychologists and physicians visited participants at home and conducted structured clinical interviews. Sociodemographic variables were assessed by a standardized questionnaire and included, among others, age, sex, education, marital status, and living situation.

### Cognitive Function

The Mini-Mental State Examination (MMSE) scores provided a measure for change in cognitive function (28). The MMSE consists of 11 questions and activities regarding, e.g., orientation, recall, and visual construction. Higher scores indicate better overall cognitive function. The maximum score is 30. For analyses, we transformed raw total MMSE scores into normalized MMSE scores (range 0–100) based on work proposed by Philipps et al. (29). The MMSE was administered as part of the structured interview for the diagnosis of dementia of Alzheimer type, multi-infarct dementia, and dementia of other etiology according to the Diagnostic and Statistical Manual of Mental Disorders (DSM), version DSM-III-R and DSM-IV, and International Statistical Classification of Diseases and Related Health Conditions (ICD), version ICD-10 (SIDAM) (30).

### Dementia Diagnosis

The SIDAM interview was used to identify dementia cases in the AgeCoDe/AgeQualiDe cohort. Incident dementia cases at observation onset were excluded in this study. The SIDAM was specifically designed to diagnose dementia according to the above named criteria. It contains a) a neuropsychological test battery (largely comprising the MMSE), b) a 14-item scale for the assessment of activities of daily living (SIDAM-ADL-Scale), and c) the Hachinski Rosen-Scale (31). Dementia was diagnosed in a consensus conference with the interviewer and an experienced geriatrician or geriatric psychiatrist according to the criteria of DSM-IV, which is implemented as a diagnostic algorithm in the SIDAM.

### Social Network and Social Isolation

We administered the short form of the Lubben Social Network Scale (LSNS-6), a quantitative measure of social network size with the purpose to assess social isolation in older adults by measuring number and frequency of contacts with friends and family as well as social support received by them (11). The LSNS-6 was first implemented at follow-up 5 of the AgeCoDe/AgeQualiDe cohort and then re-assessed at each further follow-up. Each of the six LSNS-6 questions is scored from 0 to 5 and the total score ranges from 0 to 30. Higher scores indicate larger social networks. A score below 12 is considered an indicator of social isolation, which means that, on average, there are fewer than two individuals available for the aspects of social networks assessed (11). *Vice versa*, a LSNS-6 score of 12 or higher indicates social integration.

### Health Characteristics

Mobility, vision, and hearing impairment were assessed at each assessment wave in self-report using a single, self-composed question for each domain. Specifically, we asked participants “do you have any difficulty in walking/hearing/vision?,” and responses were recorded using an ordinal scale of severity: 1) no difficulty, 2) some difficulty, 3) significant difficulty, and 4) extreme difficulty or unable to walk/blind/deaf. To prevent sparsely populated cells, response options 2–4 were collapsed into a binary indicator of impairment (yes/no).

Depressive symptoms were identified with the short version of the Geriatric Depression Scale (GDS) (32). The GDS consists of 15 questions specific to older age, e.g., “have you dropped many of your activities and interests?”. The maximum score is 15 (score > 5 indicating increased depressive symptomatology; score > 10 indicating severe depressive symptomatology).

Additionally, participants’ GPs completed self-developed standardized questionnaires regarding the presence or absence of typical chronic conditions in old age at each assessment wave. In this study, we incorporated whether participants had a history of stroke, diabetes mellitus, and hypertension.

### Statistical Analysis

An α-level for statistical significance of 0.05 (two-tailed) was applied for all analyses which were performed using Stata 13.1 SE (Stata-Corp LP, College Station, TX). Descriptives of sample characteristics were calculated, and differences in sociodemographic and health characteristics at baseline were inspected in socially isolated (LSNS-6 < 12) *vs.* socially integrated (LSNS-6 ≥ 12) individuals using *t*-tests for continuous variables and Chi square tests (*χ²)* for categorical variables.

We used linear hybrid mixed effects regression models to analyze longitudinal effects of social network size on cognitive function. Hybrid models allow for disentangling effects of time-variant within-subject and time-invariant between-subject effects of risk factors on cognitive function (33). Resulting within- and between-subject effects can be interpreted independently, combining the strengths of random- and fixed-effects models (34). Random effects are analyzed for time-invariant variables (age at observation onset, sex, education, history of stroke/hypertension/diabetes mellitus). Time-varying variables (social network size, marital status, living situation, mobility and sensory impairment, depressive symptoms) can be differentiated between within-estimators and between-estimators. Scale scores (social network size/LSNS-6 scores, age at observation onset, cognitive function/normalized MMSE scores, depressive symptoms/GDS scores) were implemented as continuous variables, all others as binary categorical variables. Three different models were defined: a) a model adjusting for key sociodemographic variables (age, sex, education) only to estimate the mere association of social network size and MMSE scores, b) a model adjusting for further sociodemographic and health characteristics that may confound the association of social network size and MMSE scores, and c) a model further adjusting for history of stroke, hypertension, and diabetes, which was performed separately due to a large number of unavailable information on comorbidity.

### Sensitivity Analysis

We reran all analyses excluding cases of MCI at observation onset to investigate effects in cognitively unimpaired individuals. Diagnosis of MCI followed Winblad criteria (35): i) no dementia according to DSM-IV, ii) minimal impairment in instrumental activities of daily living (IADL) as assessed by the SIDAM-ADL-scale, iii) evidence of cognitive decline in self- or informant-report and in objective cognitive tests (i.e., positive response to “do you feel as if your memory is becoming worse?” and test performance of one standard deviation below age- and education-specific norms on at least one main domain of cognitive function as assessed by the SIDAM incl. MMSE items).

## Results

### Sample Characteristics

The sample of *n* = 942 dementia-free individuals had a mean age of 86.4 years (*SD* = 3.0, range = 81–97), 68.2% were women. Social isolation was prevalent in one third of the sample (32.3%). The total follow-up time was 4.7 years. The average follow-up time was 3.5 years. Altogether, n = 584 (62.0%) individuals completed all five assessment waves up to follow-up 9. Individuals who were lost to follow-up did not differ regarding sex [females: 65.9% *vs.* 69.5%; *X*^*2*^(1) = 1.32, *p* = .250] or education (high level of education: 12.0% *vs.* 14.0%; *X*^*2*^(1) = 0.80, *p* = .373) compared to completers, however they were more often socially isolated [37.2% *vs.* 29.3%; *X*^*2*^(1) = 6.29, *p* = .012], older (mean age: 86.8 *vs.* 86.1 years; *t*(940) = 3.63, *p* < .001), and had lower MMSE scores (mean scores: 27.7 *vs.* 28.1; *t*(940) = −3,57, *p* < .001) than completers.

The average social network size (LSNS-6 score) was *M* = 8.1 (*SD* = 2.7) in socially isolated participants *vs. M* = 17.3 (*SD* = 3.9) in socially integrated individuals [*t*(940) = 37.48, *p* <.001; *d* = −2.76]. Socially isolated individuals were significantly older, less often married/in a partnership, and more often living alone than socially integrated individuals. Moreover, they had lower MMSE scores, were more frequently impaired in mobility and vision, and had higher depressive symptoms. However, socially isolated individuals did not differ from socially integrated individuals regarding sex, education, history of stroke, diabetes, and hypertension, as well as hearing impairment. Sample characteristics are detailed in **Table 1**.

**Table 1:** Sociodemographic and health characteristics of the study sample at observation onset.

Variable	Total (*n* = 942)	Socially isolated individuals*(*n* = 304)	Socially integrated individuals* (*n* = 638)	*p-*Value	Effect size
Age, *M* (*SD*)	86.37 (2.98)	86.90 (3.06)	86.12 (2.90)	<.001	*d* = 0.26
Sex, *n* (%)					
Female	642 (68.2)	218 (71.7)	424 (66.5)	.106	
Male	300 (31.8)	86 (28.3)	214 (33.5)		
High education, *n* (%)	125 (13.3)	36 (11.8)	89 (13.9)	.373	
Social network size (LSNS-6), *M* (*SD*)	14.32 (5.58)	8.07 (2.73)	17.30 (3.86)	<.001	*d* = −2.76
Married/in partnership, *n* (%)	280 (29.8)	65 (21.5)	215 (33.7)	<.001	*Φ* = 0.13
Living alone, *n* (%)	497 (52.8)	319 (50.0)	178 (58.6)	.014	*Φ* = 0.08
Cognitive function (MMSE), *M* (*SD*)	27.97 (1.72)	27.66 (1.87)	28.12 (1.62)	<.001	*d* = −0.26
History of, *n* (%)					
Stroke	42 (5.8)	14 (6.0)	28 (5.7)	.901	
Diabetes mellitus	197 (27.4)	68 (28.9)	129 (26.5)	.520	
Hypertension	618 (84.7)	199 (84.3)	417 (84.9)	.831	
Mobility impairment, *n* (%)	538 (57.1)	202 (66.4)	336 (52.7)	<.001	*Φ* = 0.13
Hearing impairment, *n* (%)	456 (48.4)	150 (49.3)	306 (48.0)	.692	
Vision impairment, *n* (%)	236 (25.1)	90 (29.6)	146 (22.9)	.026	*Φ* = 0.07
Depressive symptoms (GDS), *M* (*SD*)	2.53 (2.51)	3.45 (2.88)	2.09 (2.19)	<.001	*d* = 0.53

GDS, Geriatric Depression Scale (score range: 0–15); LSNS-6, short form of the Lubben Social Network Scale (score range = 0–30); M, mean; MMSE, Mini-Mental State Examination (score range: 0–30); SD, standard deviation; missing values: married/in partnership: n = 1 (0.1%); stroke: n = 218 (23.1%); diabetes mellitus: n = 223 (23.7%); hypertension: n = 215 (22.8%); depressive symptoms: n = 7 (0.7%);

*Based on the total score of the short form of the Lubben Social Network Scale (LSNS-6, scoring range = 0–30), which defines social isolation as a score below 12 and social integration as a score equal 12 or higher.

### Effects of Social Network Size on Cognitive Function

**Table 2** presents the results of the linear hybrid regression models. Across all models, social network size had both a significant between and within effect on cognitive function. Specifically, changes in an individual’s social network size were associated with changes in cognitive function over a mean follow-up time of 3.5 years (within effect). An individual’s one point change in the LSNS-6 score was associated with an average *β* = 0.2 (95% CI = 0.1; 0.4; *p* = .003) change in the normalized MMSE score, taking sociodemographic and health characteristics into account (model 3 in **Table 2**). Shrinking social network size was thus associated with decreasing cognitive function. Likewise, individuals with smaller social networks had lower cognitive function compared to individuals with larger social networks (between effect). Each point difference on the LSNS-6 scale between participants was associated with *β* = 0.5 (95% CI = 0.3; 0.7; *p* < .001) difference in cognitive scores, indicating better cognitive function in individuals with larger social networks.

**Table 2:** Adjusted hybrid regression effect estimates for cognitive function (normalized MMSE scores) over time in dementia-free oldest-old individuals (*n* = 942).

	Model 1			Model 2			Model 3		
	Coef. (95% CI)	*SE*	*p*-value	Coef. (95% CI)	*SE*	*p*-value	Coef. (95% CI)	*SE*	*p*-value
**Random effects**									
Age at observation onset*	−0.60 (−0.92; −0.27)	0.17	<.001	−0.51 (-.82; −0.19)	0.16	.001	−0.44 (−0.80; −0.07)	0.18	.018
Male sex	−1.21 (−3.22; 0.81)	1.03	.241	−1.24 (−3.48; 1.00)	1.14	.227	−-0.66 (−3.14; 1.81)	1.26	.599
High education	5.83 (3.07; 8.59)	1.41	<.001	6.05 (3.58; 8.52)	1.26	<.001	5.69 (3.08; 8.29)	1.33	<.001
History of									
Stroke							−1.51 (−6.71; 3.69)	2.65	.568
Diabetes mellitus							−1.72 (−3.79; 0.35)	1.06	.103
Hypertension							0.28 (−2.39; 2.94)	1.36	.840
**Within-estimators**									
Social network size*	0.50 (0.35; 0.64)	0.08	<.001	0.26 (0.13; 0.40)	0.07	<.001	0.23 (0.08; 0.38)	0.08	.003
Married/in partnership (ref. single, widowed)				4.70 (1.67; 7.72)	1.54	.002	4.17 (0.95; 7.40)	1.64	.011
Living alone (ref. shared housing)				5.07 (2.99; 7.15)	1.06	<.001	5.32 (2.96; 7.68)	1.20	<.001
Mobility impairment (ref. no impairment)				−1.61 (−3.05; −0.17)	0.73	.028	−1.34 (−2.97; 0.29)	0.83	.108
Hearing impairment (ref. no impairment)				−0.65 (−1.68; 1.42)	0.79	.872	0.74 (−1.05; 2.53)	0.91	.416
Vision impairment (ref. no impairment)				−1.46 (−2.88; −0.04)	0.72	.044	−1.24 (−2.82; 0.33)	0.81	.123
Depressive symptoms*				−0.65 (−0.97; −0.33)	0.16	<.001	−0.75 (−1.12; −0.38)	0.19	<.001
**Between-estimators**									
Social network size*	0.77 (0.56; 0.98)	0.11	<.001	0.50 (0.31; 0.70)	0.10	<.001	0.51 (0.29; 0.74)	0.11	<.001
Marital status				3.30 (0.18; 6.42)	1.59	.038	4.33 (0.73; 7.92)	1.83	.018
Living alone				5.71 (3.11; 8.30)	1.32	<.001	7.17 (4.06; 10.28)	1.59	<.001
Mobility impairment				−0.15 (−2.59; 2.29)	1.24	.902	0.85 (−1.95; 3.64)	1.43	.554
Hearing impairment				0.15 (−1.88; 2.18)	1.04	.884	−0.39 (−2.68; 1.91)	1.17	.742
Vision impairment				1.87 (−0.60; 4.33)	1.26	.138	1.59 (−1.16; 4.35)	1.41	.256
Depressive symptoms*				−0.29 (−0.71; 0.14)	0.22	.183	−0.25 (−0.73; 0.23)	0.24	.302

*Continuous scores, estimates display average change in cognitive function per one unit increase in scores; 95% CI , 95% confidence interval; MMSE, Mini-Mental Status Examination; SE, standard error.

### Random Effects of Time-Invariant Covariates

Subsequently, we present results of the fully adjusted model (model 3) only. All other estimates are detailed in **Table 2**. Higher age at baseline was associated with lower cognitive function (unit change per one more year of age: *β* = −0.4, 95% CI = −0.8; −0.1; *p* = .018). Higher education showed a protective effect on cognitive function (contrasted with middle/low education: *β* = 5.7, 95% CI = 3.1; 8.3; *p* < .001). There was no significant effect of sex (ref. female: *β* = −0.7, 95% CI = −3.1; 1.8; *p* = .599). Neither a history of stroke (*β* = −1.5, 95% CI = −6.7; 3.7; *p* = .568), diabetes mellitus (*β* = −1.7, 95% CI = −3.8; 0.3; *p* = .103), or hypertension (*β* = 0.3, 95% CI = −2.4; 2.9; *p* = .840) was associated with cognitive function.

### Within Effects of Time-Variant Covariates

Besides social network size, changes in marital status, living situation, and depressive symptoms independently had a significant longitudinal effect on cognitive function. Changes in partnership status were associated with changes in cognitive function with remaining in a partnership or marriage (ref. being single/widowed: *β* = 4.2, 95% CI = 1.0; 7.4; *p* = .011) as well as living alone (ref. shared housing: *β* = 5.3, 95% CI = 3.0; 7.7; *p* < .001) showing a protective effect. Increases in depressive symptoms were associated with decreases in cognitive function (unit change per each point difference on the GDS scale: *β* = −0.7, 95% CI = −3.1; 1.8; *p* < .001). Changes in mobility, hearing, and vision were not associated with changes in cognitive function.

### Between Effects of Time-Variant Covariates

Individuals in a marriage or partnership showed better cognitive function as opposed to single or widowed individuals (*β* = 4.3, 95% CI = 0.7; 7.9; *p* = .018). Individuals who were living alone showed better cognitive function as opposed to individuals who shared housing (*β* = 7.2, 95% CI = 4.1; 10.3; *p* < .001). There were no significant between effects regarding individuals with or without mobility impairment, hearing impairment, vision impairment, and depressive symptoms.

### Sensitivity Analysis

When rerunning all models excluding individuals with a diagnosis of MCI at observation onset (*n* = 109; 11.6%), i.e., inspecting individuals without cognitive impairment only, results were similar. Across all models, social network size had both a between effect and a within effect on cognitive function in oldest-old individuals without cognitive impairment. Taking sociodemographic and health characteristics into account, an individual’s one point change in the LSNS-6 score was associated with an average *β* = 0.2 (95% CI = 0.1; 0.4; *p* = .006) change in the normalized MMSE score. Moreover, each point difference on the LSNS-6 scale between participants was associated with *β* = 0.5 (95% CI = 0.2; 0.7; *p* < .001) difference in cognitive scores, indicating better cognitive function in individuals with larger social networks. Results of the sensitivity analysis are not further shown.

## Discussion

We aimed to longitudinally investigate between and within effects of social network size on cognitive function in a large sample of dementia-free oldest-old individuals. Social isolation was highly prevalent in our sample (32.3%). In line with our hypotheses, social network size was consistently associated with cognitive function across all analyses. Oldest-old individuals with smaller social networks showed lower cognitive function than individuals with larger social networks. Moreover, changes in individuals’ social network sizes were associated with changes in cognitive function, indicating decreasing cognitive function with shrinking social networks. This was the case independently of marital status and living situation as well as above and beyond effects of age, sex, education, and health characteristics. Our results therefore emphasize the relevance of the social network for cognitive function in the segment of the oldest-old age group, of which one third was socially isolated. A novel aspect of our work is that our results show that both an individual’s social network size *per se* and changes in an individual’s social network size are associated with cognitive function.

In general, it is not well known whether or not certain lifestyle factors, including social isolation, are related to cognitive function in the oldest-old (36). In fact, lifestyle associations with cognitive function and dementia may present differently in midlife *vs.* younger-old age *vs.* older-old age (19, 26). Therefore, it is important to study lifestyle factors and cognitive function in regard to different age groups. It has relevant implications for shaping prevention and intervention regimes.

In line with a few previous studies, we provide further evidence that social network size is associated with cognitive function in oldest-old age (23–25). This highlights the relevance of maintaining a socially active lifestyle into latest life as it might protect against cognitive decline (37). However, this may be challenging as decreases in social network size with increasing age are inevitable to a certain extent. This is for varying reasons, including migration of children, other relatives, and friends as well as death or disability of social network members (38). Death and disability of social network members of similar age (e.g., spouse, siblings, friends, and colleagues) is a natural explanation for increasing social isolation with aging, with little to do about. However, increasing migration of family can be considered a contemporary (period) effect that leads toward scattered and more fragile social networks limiting the options for direct social contact in the community, explaining increasing prevalence in social isolation over time. In consideration of global population aging, this development calls for urgent action to tackle social isolation.

Targeting interventions to reduce social isolation in old age is perhaps the most obvious approach to prevent or attenuate its adverse outcomes. However, studies summarizing the effectiveness of such interventions reported limited evidence, with inconsistent findings for group based interventions (39, 40) and e-interventions (41). The success may be even smaller if social isolation is accompanied by depressive symptoms, which is a common scenario (42). Likewise, participation in interventions against social isolation may not be for everybody, whether it be for mobility impairment or poor health. Particularly, the older individuals get, the more they may struggle to involve in new social relations, and sometimes even wish to disengage from society (43). Moreover, interventions against social isolation may fail to address what is important in relationships with others: meaningfulness and a sense of belonging (44).

The difficulty of delivering effective interventions against social isolation in old age draws the attention toward prevention. There are hints that promising approaches may include large-scale community initiatives, social prescribing, and improving social cognition (45, 46). Nicholson (47) suggested including the assessment of social network in older adults in routine health care to identify at-risk individuals and refer them to community resources in order to prevent social isolation or further isolation, which could in turn reduce its negative health outcomes.

The high prevalence of social isolation and its negative effects on cognitive function in oldest-old age make it a crucial factor to target for dementia prevention. We argue that social isolation could be a moderator or mediator for other modifiable lifestyle-related risk factors for dementia, e.g., physical activity and diet. Studies have shown that social support is very important for adherence to lifestyle programs (48). Social isolation in old age could thus be a hindering factor in leading a healthy lifestyle that promotes brain health. Future studies investigating this assumption are required.

### Limitations

Among the study’s limitations is first to mention that the generalizability of results might be limited because of a moderate response rate of individuals to the study and a substantial number of subjects who could not be located or refused participation in follow-up assessments which biases our analytical sample towards healthier subjects. Thus, our results could be an underestimation of effects.

Moreover, our measure for social network size, the LSNS-6, does not capture qualitative aspects of social network, such as perceived isolation and loneliness that may impact health differently, although the literature also describes an association of perceived isolation and loneliness with cognitive function (49). In addition, social network size masks other qualitative aspects as well, e.g., social relationships can be toxic and less a source of support, which therefore may not be beneficial for cognitive health. This needs further study. We were also not able to address other potentially confounding factors, for example, social activities, personality traits, or attachment styles as these variables were not collected in AgeCoDe/AgeQualiDe.

Furthermore, the MMSE may not be the ideal instrument to assess cognitive function in non-demented populations as it has been shown that scores can exhibit ceiling effects or limited sensitivity regarding subtle cognitive impairment. To this end, our results should be interpreted with caution as they are likely conservative estimates, potentially underestimating the extend of the association with social network size.

Last, it is necessary to discuss that social isolation can also be prodromal to dementia as individuals who notice deterioration in cognitive function may withdraw from social activities (50). Moreover, it is common that individuals who receive a diagnosis of dementia are avoided in their social life (51). However, we conducted a sensitivity analysis with cognitively unimpaired oldest-old individuals and results further suggested an adverse association of smaller social networks and cognitive function, supporting the notion of social isolation being a risk factor for cognitive decline and dementia. However, the relationship of limited social networks or social isolation and cognitive function is rather reciprocal. Nonetheless, our findings from a substantial sample of oldest-old individuals are sufficient to provide further evidence for a robust association of social network size and cognitive function in the oldest-old.

## Conclusion

Social isolation is highly prevalent among oldest-old individuals. Smaller social networks and shrinking social networks were longitudinally associated with lower cognitive function in this age group. Our findings demonstrate the importance of maintaining a socially active lifestyle for brain health in oldest-old age. However, as social network size decreases and risk for social isolation increases with aging, it is important to find effective ways to intervene against, or even better, to prevent social isolation. Considering global population aging, the high prevalence of social isolation in old age as well as its adverse consequences for health, social isolation should be a public health priority.

## Data Availability Statement

The dataset is available for research purposes upon reasonable request to the data handling center of the AgeCoDe/AgeQualiDe study. Please inquire to BW, Wiese.Birgitt@MH-Hannover.de.

## Ethics Statement

The studies involving human participants were reviewed and approved by Ehtics committee of the Medical Faculty of the University of Leipzig. The patients/participants provided their written informed consent to participate in this study.

## Author Contributions

Study concept and design: SR, WM, MS, MW, SR-H. Acquisition of data: SR, ML, UG, KH, MP, AF, ME, HK, H-HK, CB, BW, SM, SW, JW, HB, DW, WM, MS, MW, SR-H. Analysis and interpretation of data: SR, LK, MW, SR-H. Drafting the manuscript: SR. Critical revision of the manuscript for important intellectual content: all authors. Final approval of the version to be published: all authors.

## Funding

This study is part of the German Research Network on Dementia (KND), the German Research Network on Degenerative Dementia (KNDD; German Study on Aging, Cognition and Dementia in Primary Care Patients; AgeCoDe), and the Health Service Research Initiative (Study on Needs, health service use, costs and health-related quality of life in a large sample of oldest-old primary care patients (85+; AgeQualiDe)) and was funded by the German Federal Ministry of Education and Research (grants KND: 01GI0102, 01GI0420, 01GI0422, 01GI0423, 01GI0429, 01GI0431, 01GI0433, 01GI0434; grants KNDD: 01GI0710, 01GI0711, 01GI0712, 01GI0713, 01GI0714, 01GI0715, 01GI0716; grants Health Service Research Initiative: 01GY1322A, 01GY1322B, 01GY1322C, 01GY1322D, 01GY1322E, 01GY1322F, 01GY1322G). The study is published in affiliation with the study “Healthy Aging – gender specific trajectories into latest life (AgeDifferent.de)” that was funded by the German Federal Ministry of Education and Research (BMBF; grant numbers: 01GL1714A, 01GL1714B, 01GL1714C, 01GL1714D). This paper was further supported by a grant from the Hans and Ilse Breuer Foundation.

## Conflict of Interest

The authors declare that the research was conducted in the absence of any commercial or financial relationships that could be construed as a potential conflict of interest.
